# Evaluating the Impact of a One‐Year Exercise Program on Probable Sarcopenia in Older Adults

**DOI:** 10.1155/jare/5534766

**Published:** 2026-06-15

**Authors:** Emanuele Marzetti, Alejandro Álvarez-Bustos, Samuel da Silva Aguiar, Ivan de Oliveira Gonçalves, Isabel Rodríguez-Sánchez, Helio José Coelho-Junior

**Affiliations:** ^1^ A. Gemelli University Hospital Foundation (IRCCS), Rome, Italy; ^2^ Department of Geriatrics, Orthopedics and Rheumatology, Catholic University of the Sacred Heart, Rome, Italy, unicatt.it; ^3^ Department of Health, Villanueva University, Madrid, Spain; ^4^ Post-Graduation in Genomic and Biotechnology Sciences, Center for Proteomic and Biochemical Analysis, Catholic University of Brasilia, Federal District, Brasília, Brazil, ucb.br; ^5^ Postgraduate Program in Physical Education, Catholic University of Brasília, Brasília, Brazil, ucb.br; ^6^ Center for Therapeutical Exercise, São Paulo, Brazil; ^7^ Hospital Clínico San Carlos, Health Research Institute, Madrid, Spain, madrid.org

**Keywords:** exercise training, mobility, muscle power, muscle strength

## Abstract

**Objectives:**

The present study was conducted to examine the effects of a 1‐year multicomponent exercise training (MCET) program on the physical function and sarcopenia status of community‐dwelling older adults with probable sarcopenia.

**Methods:**

Data of 132 Brazilian community‐dwelling older adults with probable sarcopenia were examined. The MCET program was performed twice a week over 1 year. Physical performance evaluations included (i) timed “up‐and‐go” (TUG), (ii) one‐leg stand, (iii) walking speed (WS) at normal pace and fast pace, (iv) 5‐time sit‐to‐stand (5STS) test, and (v) isometric handgrip strength (IHG).

**Results:**

MCET significantly increased TUG (−7.0%), balance (53.0%), 5STS (−10.0%), and WS at normal pace (13.0%). Furthermore, a significant decrease in the prevalence of IHG‐based probable sarcopenia was observed (88.6% vs. 67.4%).

**Conclusions:**

Findings of the present study indicate that a 1‐year intervention program based on an MCET strategy significantly improved multiple physical performance measures and enhanced sarcopenia status in community‐dwelling older adults. These improvements may significantly lower the risk of developing adverse outcomes, such as falls, disability, hospitalization, and death. Therefore, the MCET program tested in this study shows promise as a primary care strategy to reduce the burden of physical decline in community‐dwelling older adults.

## 1. Introduction

The aging process is often associated with gradual impairments in various physical capabilities [1–4]. In the last decades, considerable attention has been given to losses of muscle strength, given its independent association with more severe clinical outcomes, including falls, disability, and death [5–8]. The importance of this scenario was recognized by international working groups, which described this condition as a state of probable sarcopenia [9].

Sarcopenia is a neuromuscular disease characterized by simultaneous losses in muscle strength and mass, with detriments in physical performance examined to evaluate its severity [9]. Although there is substantial discussion on the most effective diagnostic method for efficiently detecting muscle failure and predicting clinical outcomes [10, 11], numerous studies have supported the premises that sarcopenia is highly prevalent in older adults and associated with many health events.

Indeed, the global prevalence of sarcopenia is estimated to range from 8% to 36%, with variations occurring in function of the identification method [12]. Notably, older adults in South America appear to be particularly vulnerable to sarcopenia, with prevalence rates reaching up to 35% in this population [12]. This information deserves attention because sarcopenia is associated with numerous conditions, including cognitive function, blood pressure levels, glucose regulation, depressive symptomatology, and dementia, and quality of life [13–17], while it was demonstrated to be a good predictor of many others (e.g., falls, disability, and death) [18, 19]. Such a scenario requires pharmacological therapy, specialized care, and hospitalization, among other factors, which help explain the significant economic impact of sarcopenia on healthcare systems [20].

Nevertheless, age‐related neuromuscular impairments are not restricted to muscle strength and commonly involve many other physical capacities, including muscle power, mobility, and balance, to quote a few [1–3]. These declines are also significantly associated with increased risk of adverse events [21–26], suggesting that the failure to maintain physical performance in older adults has a cumulative effect on overall health and well‐being. Therefore, the early and effective management of physical decline should be a priority of public health programs aimed at preserving older adults’ independence and health status [27, 28], promoting successful aging [29, 30].

Exercise training is widely acknowledged as a primary or complementary therapy for managing chronic conditions in older adults [31–33]. Among the variety of exercise regimes, multicomponent exercise training (MCET) stands out for its ability to simultaneously promote gains in different physical aspects [34, 35]. Many studies have confirmed these assumptions by indicating that interventions based on MCET might significantly improve physical performance in older adults [36–41].

However, this body of evidence is based on apparently healthy community‐dwelling individuals or those with severe stages of physical decline (e.g., frailty), while to the best of our knowledge, no studies have examined people with probable sarcopenia. Moreover, most studies are based on short‐term programs (e.g., 12 weeks) [41], which limit a comprehensive understanding of their possible role as strategies for public health.

Based on these premises, the present study was conducted to examine the effects of a 1‐year MCET intervention on the physical function and sarcopenia status of community‐dwelling older adults with probable sarcopenia.

## 2. Materials and Methods

### 2.1. Study Design

This quasi‐experimental study evaluated the effects of a 1‐year MCET program on physical function and sarcopenia status in community‐dwelling older adults with probable sarcopenia. The study protocol was approved by the Research Ethics Committee of the University of Mogi das Cruzes (01550237000‐10, date: 22 April 2014) and conducted in accordance with the principles of the Declaration of Helsinki (1964, and subsequent revisions: 1975, 1983, 1989, 1996, and 2000) and Resolution 196/96 of the Brazilian National Health Council. Reporting of the study followed the Transparent Reporting of Evaluations with Nonrandomized Designs (TREND) guidelines, applying relevant items as appropriate.

### 2.2. Participants

Participants were drawn from the Cantinho do Idoso da Cidade de Poá (CICP) cohort [42–44]. This study, initiated in 2013 by Prof. Helio Jose Coelho Junior, Prof. Samuel da Silva Aguiar, and Prof. Ivan de Oliveira Gonçalves, aimed to examine, describe, and assess the physical performance status of community‐dwelling older adults attending a senior center in Poá, São Paulo, Brazil. Evaluations were conducted every 6 months until 2019. Waves are being matched, and data of the first data collections (i.e., 2014‐2015) have been examined. No changes in the data collection protocol occurred in the waves analyzed in the present study.

Participants were recruited using convenience sampling, whereby eligible individuals were selected based on their availability and willingness to participate rather than through random selection. No formal sample size calculation was performed. Participants were verbally invited by physicians and research staff during routine clinical visits. Eligibility criteria included the following: (a) age ≥ 60 years, (b) residence in the community, (c) independence in basic activities of daily living according to the Katz Index (score = 6) [45], (d) ability to walk independently without assistive devices, (e) absence of dementia, and (f) provision of written informed consent. Exclusion criteria included changes in medication during the intervention period, the presence or development of physical (e.g., angina) or psychological (e.g., fear) symptoms that caused discomfort during exercise, diagnosed pulmonary, neurological, or psychiatric disorders (e.g., Parkinson’s disease and Alzheimer’s disease), musculoskeletal disorders, recurrent dizziness, blurred vision, orthostatic intolerance, and absence from more than three exercise sessions. Participants who were receiving or initiated hormone replacement therapy or psychotropic medications during the study period were also excluded.

For this analysis, only individuals classified as having probable sarcopenia—based on reduced isometric handgrip strength (IHG) and/or poor performance on the five‐time sit‐to‐stand (5STS) test, according to the cutoff points proposed by the European Working Group on Sarcopenia in Older People 2 (EWGSOP2) [9]—and with complete data in the 2014‐2015 evaluations were included.

### 2.3. Physical Function Evaluation

Physical performance evaluations included (i) timed “up‐and‐go” (TUG), (ii) one‐leg stand, (iii) walking speed (WS) at normal pace and fast pace, (iv) 5STS test, and (v) IHG [1]. All physical function assessments were conducted by two experienced exercise physiologists. One examiner explained the testing procedures, demonstrated each test prior to assessment, recorded performance outcomes, and observed movement patterns. The second examiner focused on participant safety, offering verbal or tactile cues when necessary, without influencing test performance. Following the instructions and before each assessment, participants completed a familiarization trial to confirm their understanding of the procedures. Then, tests were performed twice, except for the one‐leg stand.

#### 2.3.1. TUG

The TUG test required participants to rise from a chair (overall height: 87 cm; seat height: 45 cm; width: 33 cm), walk 3 m around a floor marker, return to the starting point, and sit back down [46, 47]. Twenty‐two participants completed the task wearing their usual shoes, with their backs resting against the backrest, arms placed on the armrests, and feet positioned on the ground. At the researcher’s signal (“go”), they stood up, walked 3 m at their fastest safe pace, turned around the cone, walked back, and sat down. The stopwatch started as soon as the participant rose from the chair and stopped once their back contacted the chair’s backrest again. The test reliability in the present study was 0.8 or more (*κ* = 0.93).

#### 2.3.2. One‐Leg Stand

Participants stood on their dominant leg while bending the opposite knee to 90°. Arms were crossed over the chest and the head kept upright. Timing began when the nondominant foot lifted off the floor and ended when it touched the ground again [48]. The test was capped in 30 s.

#### 2.3.3. WS at Normal Pace and Fast Pace

WS was measured over a central 3‐m distance within a 5‐m course (including 1‐m acceleration and deceleration zones). Participants walked at their usual pace, followed by their fastest safe pace. Timing began when a foot crossed the 1‐m line and stopped when it reached the 4‐m line [26, 49]. The test reliability in the present study was 0.8 or more (*κ* = 0.98).

#### 2.3.4. 5STS

Participants stood up and sat down five times as quickly as possible, with arms crossed over the chest. Timing started when they lifted off the chair and stopped at the end of the fifth repetition [50, 51]. A 5STS time exceeding 15 s was used to identify participants with probable sarcopenia [9]. The test reliability in the present study was 0.8 or more (*κ* = 0.97).

#### 2.3.5. IHG

IHG was assessed using a Jamar hydraulic dynamometer (Sammons Preston, Bolingbrook, IL, USA). Participants were seated with the shoulder slightly abducted, elbow at 90°, and wrist in a neutral position. Each participant performed one maximal contraction of 4 seconds using their dominant hand, identified by self‐report [6, 7]. A IHG result < 27 kg, for men, and < 16 kg, for women, was used to identify participants with probable sarcopenia [9]. The test reliability in the present study was 0.8 or more (*κ* = 0.97). Results were recorded in kilograms.

### 2.4. MCET

The MCET was developed in accordance with the framework proposed by Tarazona‐Santabalbina et al. [52], which integrates endurance, strength, balance, coordination, and flexibility exercises. Sessions lasted approximately 60 min, were held twice weekly on nonconsecutive days, and continued for 1 year under the supervision of qualified exercise physiologists at the Centro de Convivência do Idoso (CCI) in Poá, Brazil.

Each session comprised 3‐4 rounds of 13 exercise stations targeting resistance, balance/proprioception, coordination, flexibility, and gait (endurance). Examples of exercises used in the MCET protocol are provided in Table 1.

**TABLE 1:** Examples of exercises used in the MCET program.

Resistance training	Balance/proprioception	Coordination	Flexibility	Gait tasks
*Lower-limb exercises*				
Squat in the chair	One‐leg stand	Ankle and hand circles	Sit and reach	Walk at normal pace
Knee extension	Tandem feet	Opposites up	Seated knee‐to‐chest	Walk at fast pace
Knee flexion	Semi‐tandem feet	Side tap swap	Hamstrings and calf stretch	Heel‐to‐toe walk
Hip abduction/adduction	Balance with wobble boards			Stepping over obstacles
Hip flexion				Walking around obstacles
Hip extension				
Calf raise				
Seated crunch				
Pelvic tilt				

*Upper-limb exercises*				
Push‐ups in the wall				
Shoulder press				
Side lateral raise				
Front raise				
Row				
Biceps curl				

Specifically, six stations targeted resistance (five lower‐limb and one upper‐limb), four emphasized balance/proprioception, two focused on coordination, and one addressed flexibility. Each station was performed for 1 min, while gait activities lasted 2 min. This structure was based on the premise that stimulating muscle strength, mobility, and balance is essential for maintaining or improving neuromuscular function, thereby supporting functional independence and potentially reducing the risk of sarcopenia and frailty [53–57].

**FIGURE 1:**
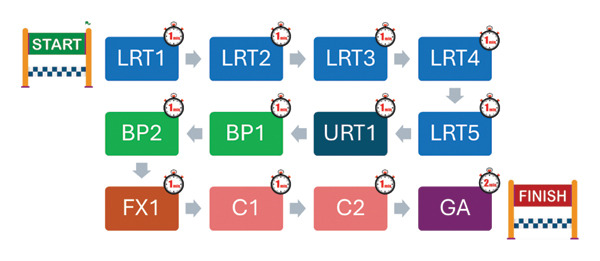
Schematic representation of one set of exercises used in the MCEP.

A schematic representation of one set of exercises used in the MCET is shown in Figure 1.

Health professionals responsible for exercise prescription are expected to select one of the physical exercises proposed in Table 1 according to the target stimulus. For instance, squats in the chair, knee extensions, knee flexions, hip flexions, and calf raises may be used for lower‐limb resistance exercises (LRT1‐5). Push‐ups against the wall may be used as upper‐limb resistance exercises (URT1). Two balance and proprioception (BP1‐2) exercises could include semi‐tandem feet and balance on a wobble board. Flexibility (FX) may be stimulated with seated and reach exercises. For coordination (C), providers may select from ankle and hand circles, opposites up, and side tap swaps. Finally, gait exercise (G) involves walking at a fast pace. This series may be repeated 3‐4 times, depending on time availability. The same exercises are used throughout the session, with a different stimulus provided in the next exercise session.

Exercise intensity was monitored with the rating of perceived exertion (RPE) scale (adapted Borg CR‐10) [58]. The scale ranges from 0 (“rest”) to 10 (“maximal effort”) and includes eight descriptive anchors (very, very easy; easy; moderate; somewhat hard; hard; very hard). Participants were instructed to maintain an exertion level between 3 and 5—corresponding to moderate (3), somewhat hard (4), and hard (5)—during functional and resistance exercises, excluding balance tasks. To facilitate self‐monitoring, a large RPE chart (4‐m high × 1.3‐m wide) was prominently displayed in the training area.

Progressive overload was implemented by adjusting exercise cadence and, for resistance exercises, by incorporating elastic bands (EXTEX Sports, São Paulo, Brazil) or dumbbells to achieve the prescribed exertion range.

### 2.5. Statistical Analysis

Normality of data was tested using the Kolmogorov–Smirnov test. Continuous variables were expressed as means ± standard deviations (SDs), and categorical variables were expressed as absolute frequencies and percentages. Changes in the prevalence of probable sarcopenia, a dichotomous outcome measured at two time points, were analyzed using McNemar’s test, which is appropriate for paired nominal data. Nonparametric alternatives such as the Wilcoxon signed‐rank test were not applied, as they are intended for ordinal or continuous variables rather than binary outcomes. A two‐tailed *p* value <  0.05 was considered statistically significant. All statistical analyses were conducted using SPSS Statistics, Version 23.0 (IBM Corp., Chicago, IL, USA).

## 3. Results

### 3.1. Characteristics of Study Participants

No adverse events occurred during the exercise sessions or during evaluations. The subjects were not absent for more than three sessions of physical exercise. The adherence to the physical exercise program was 100% (0 dropouts). The main characteristics of the 132 study participants are presented in Table 2. The majority were men (61.3%), and the average age was 67.0 ± 5.8 years, indicating that the sample consisted of relatively young community‐dwelling older adults. Indeed, nearly 90% of the study participants (*n* = 118) aged 74 or less years. Mean BMI values indicate that most participants had normal weight/overweight. This tendency was confirmed when participants were examined according to BMI distribution, with most of them being classified as normal weight and overweight, some as obese, and two as underweight. Probable sarcopenia was primarily based on low IHG values (88.6%), while low 5STS performance was found in 11.4% of the examined sample. Average physical performance in all assessment tests was lower than normative values of the Brazilian population [1].

**TABLE 2:** Main characteristics of study participants (*n* = 132).

Variable	Mean ± SD (%)
Age (years)	67.02 ± 5.86
BMI (kg/m^2^)	26.50 ± 5.28
BMI distribution (*n*, %)	
Underweight (≤ 18.5 kg/m^2^)	2, 2.2
Normal weight (18.5–24.9 kg/m^2^)	44, 43.9
Overweight (25.0–29.9 kg/m^2^)	55, 55.0
Obese (≥ 30 kg/m^2^)	31, 30.7
Sex (men)	61.3%
Balance (s)	14.39 ± 12.28
TUG (s)	7.33 ± 1.45
WS at normal pace (s)	1.27 ± 0.29
WS at fast pace (s)	1.69 ± 0.58
IHG (kg)	19.65 ± 4.59
Relative IHG (kg/kg)	0.38 ± 0.28
5STS (s)	12.10 ± 3.62
IHG‐based probable sarcopenia (*n*,%)	116 (88.6)
5STS‐based probable sarcopenia (*n*,%)	16 (11.4)

*Note:* 5STS = 5‐time sit‐to‐stand (5STS) test; IHG = isometric handgrip.

Abbreviations: BMI = body mass index; TUG = timed “up‐and‐go”; WS = walking speed.

### 3.2. Effects of MCET on Physical Performance

Table 3 shows the effects of the MCET on physical performance. The exercise training program significantly increased TUG (−7.0%), balance (53.0%), 5STS (−10.0%), and WS at normal pace (13.0%). No significant effects were observed on IHG (+2%) and WS at fast pace (35%).

**TABLE 3:** Effects of MCET on physical performance.

Variable	Pre (mean ± SD)	Post (mean ± SD)
TUG (s)	7.33 ± 1.45	6.82 ± 1.62^∗^
Balance (s)	14.39 ± 12.28	22.01 ± 9.93^∗^
WS at normal pace (m/s)	1.27 ± 0.29	1.43 ± 0.25^∗^
WS at fast pace (m/s)	1.69 ± 0.58	2.04 ± 2.18
IHG (kg)	19.65 ± 4.59	20.06 ± 6.59
Relative IHG (kg/kg)	0.38 ± 0.28	0.37 ± 0.31
5STS (s)	12.10 ± 3.62	10.89 ± 5.09^∗^

*Note:* *p* < 0.05 vs. Pre. 5STS = 5‐time sit‐to‐stand (5STS) test; IHG = isometric handgrip.

Abbreviations: TUG = timed “up‐and‐go”; WS = walking speed.

^∗^ *p* < 0.05.

### 3.3. Effects of MCET on the Prevalence of Probable Sarcopenia

**FIGURE 2:**
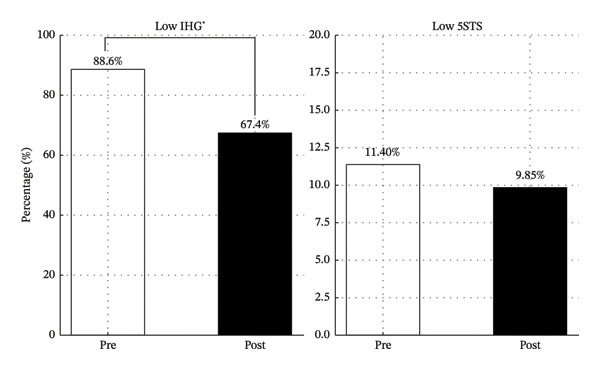
Prevalence of probable sarcopenia according to 5STS and IHG. ^∗^ *p* < 0.05 vs. Pre. Abbreviations: 5STS = 5‐time sit‐to‐stand (5STS) test; IHG = isometric handgrip.

Figure 2 shows the effects of MCET on the prevalence of probable sarcopenia, identified according to the cutoff points proposed by the EWGSOP2 [9]. A significant decrease in the prevalence of IHG‐based probable sarcopenia was observed (88.6% vs. 67.4%), while no changes were found in 5STS‐based sarcopenia (11.4% vs. 9.85%).

## 4. Discussion

The main findings of the present study indicate that a 1‐year intervention program based on a MCET strategy significantly increased physical performance in distinct assessment tests, including TUG, balance, 5STS, and WS at normal pace, among community‐dwelling older adults with probable sarcopenia. Although significant improvements in IHG performance were not observed, the exercise training program promoted a notable reduction in the prevalence of probable sarcopenia, when determined by low IHG.

To the best of our knowledge, this is the first study that examined the effects of an MCET intervention on the prevalence of probable sarcopenia. Our findings align with a substantial body of evidence supporting the effectiveness of MCET programs in the physical performance of older adults. Bird et al., [36] found that a 39‐week MCET significantly increased lower‐limb muscle strength, balance, and TUG performance of physically inactive community‐dwellers. These findings were expanded by Freiberger et al. [37], who examined the effects of a MCET program performed twice a week and composed of progressive resistance, balance, and endurance exercises. After the intervention, significant improvements in TUG, balance, 5STS, and WS at normal pace were observed [37]. More recently, Rodrigues et al. [38] confirmed these findings after investigating the impact of a MCET aligned with the recommendations of the American College of Sports Medicine (ACSM).

Similar results have been found in investigations involving individuals at comparable or subsequent stages of neuromuscular decline to those of participants of the present study. Sadjapong et al. [39] observed that a MCET involving progressive endurance, resistance, and balance training performed three times a week over 12 weeks significantly increased upper‐limb muscle strength, TUG, and balance among older adults with physical frailty, identified according to the frailty phenotype. Comparable results were found by Li et al. [40], after investigating the effects of a tailored, structured, and progressive MCET (i.e., VIVIFRAIL).

These findings have been recently confirmed in pooled analyses. For instance, Yang et al. [41] examined data of nearly five thousand frail older adults (age ranged from 62.2 to 94.3 years) from 28 studies. MCET characteristics were substantially homogenous, with intervention length varying from 12 to 52 weeks, frequency from one to seven days a week, and session duration from 26 to 90 min [41]. Findings indicated that MCET significantly improved muscle strength, WS, balance, and TUG in this population.

Our findings underscore the potential of this exercise training protocol and emphasize the need for further research using more rigorous study designs, such as randomized clinical trials. Indeed, the improvements observed in the present study might result in significant decreases in the risk of many adverse outcomes, including falls, disability, hospitalization, and death [21–26]. Moreover, the reduction in the prevalence of probable sarcopenia following the intervention suggests that older adults may be at lower risk of developing more severe stages of this condition and its numerous common outcomes [59, 60].

Nevertheless, the observation that the exercise training protocol was not sufficient to improve WS at fast pace nor reduce the prevalence of 5STS‐based probable sarcopenia indicates that this strategy might benefit from modifications to its organizational structure. Notably, both WS at a fast pace and 5STS are dependent on the ability to generate muscle strength quickly [61–64]—a physical capacity known as muscle power [65, 66].

Improvements in muscle power might be reached in response to conventional resistance training programs [67–70]. However, these outcomes are more likely to result from specific exercise programs in which concentric muscle contractions are performed as quickly as possible [68, 70]. In response to this scenario, expert consensus has endorsed power training as a valid and effective method to enhance muscle power and physical performance in older adults [71, 72]. Therefore, the absence of a specific stimulus to muscle power in the examined MCET program may have limited its impact on fast WS and 5STS.

The differences between IHG and 5STS observed at baseline and following the MCET deserve further discussion. The possibility that these physical performance measures capture distinct facets of muscle strength has been raised by research groups [73]. These premises are based on the fact that IHG and 5STS differ in biomechanical and biochemical characteristics.

In summary, IHG involves a brief maximal isometric contraction primarily of the forearm muscles, whereas the 5STS is a longer, dynamic task engaging multiple muscle groups and contraction modes [73]. These biomechanical differences likely lead to distinct metabolic demands and differing associations with other physical performance measures [73]. Overall, our findings support the view that IHG and 5STS capture different aspects of muscle function and should not be used interchangeably to identify sarcopenia, an issue that warrants further investigation [73].

Findings of the present study have important clinical implications. The characteristics of the MCEP suggest that it might be a low‐cost, effective, and safe strategy to primary care programs aimed at reducing physical decline in community‐dwelling older adults. Furthermore, the flexibility of this exercise modality allows for modifications to the program, making it adaptable to individual needs. To this end, health professionals caring for older adults should ensure a thorough evaluation of each individual’s health status.

Furthermore, from a broader perspective, emerging evidence suggests that novel care models integrating digital technologies may further enhance the management of age‐related physical decline. In this context, Kim and Kim [74] recently demonstrated that healthcare programs based on artificial intelligence and Internet of Things (AI·IoT) technologies were associated with significant improvements in health behaviors, physical activity, and cognitive function in community‐dwelling older adults after 6 months of intervention. Notably, these programs combined face‐to‐face and remote components, highlighting the potential of technology‐supported strategies to promote engagement and sustained lifestyle changes in aging populations. Although such interventions differ from structured exercise‐based approaches like MCET, they share a common goal of preserving functional capacity and independence. Future research may explore whether integrating digital health solutions with long‐term multicomponent exercise programs could further optimize outcomes in older adults with probable sarcopenia, particularly by enhancing adherence, personalization, and long‐term monitoring of physical function.

The present study has several limitations that should be acknowledged to facilitate a more accurate interpretation of our results. First, the quasi‐experimental design, characterized by the absence of randomization and a control group, may introduce selection bias and limits the ability to draw causal inferences, as observed changes cannot be attributed exclusively to the intervention. Second, key exercise variables, such as intensity and adherence, were not controlled for, which could have impacted the effectiveness of the intervention. Third, the absence of muscle mass assessment limited a more detailed evaluation of sarcopenia status. The use of assessment tools (e.g., bioelectrical impedance analysis) could have provided additional insight into the findings and should be considered in future studies. Fourth, the study did not assess nutritional status or protein intake, both of which are critical factors in muscle health and may have influenced the results. Fifth, participants were recruited for convenience, which may limit the generalizability of our findings to a broader population. Sixth, the study sample consisted of relatively young community‐dwelling individuals, mostly men, with preserved physical performance. The high adherence and complete follow‐up may be attributed to the municipally administered exercise program and the restriction on participant absences. Furthermore, these specific characteristics preclude the conduction of subgroup analysis taken into consideration the possible sex‐ and age‐related differences that might emerge in this type of analysis. As a result, caution is warranted when extrapolating these findings to other populations. Finally, the underlying mechanisms mediating the potential effects of MCET were not examined. Future studies should investigate biological factors such as inflammation, metabolism, and mitochondrial function to better understand its impact on sarcopenia [39, 75–77].

## 5. Conclusions

Findings of the present study indicate that a 1‐year intervention program based on an MCET strategy significantly improved multiple physical performance measures and enhanced sarcopenia status in community‐dwelling older adults. These improvements may substantially reduce the risk of adverse outcomes, including falls, disability, hospitalization, and mortality. Therefore, the MCET program tested in this study shows promise as a primary care strategy to mitigate physical decline in older adults. Future studies employing more robust study designs, including larger and more diverse populations, different age groups, and sex‐specific analyses, are needed to confirm these findings and clarify the underlying mechanisms of MCET on muscle function and sarcopenia prevention.

## Author Contributions

Conceptualization, Alejandro Álvarez‐Bustos, Samuel da Silva Aguiar, and Helio José Coelho‐Junior; methodology, Samuel da Silva Aguiar, Ivan de Oliveira Gonçalves, Emanuele Marzetti, and Helio José Coelho‐Junior; formal analysis, Alejandro Álvarez‐Bustos, Samuel da Silva Aguiar, Isabel Rodríguez‐Sánchez, Emanuele Marzetti, and Helio José Coelho‐Junior; investigation, Samuel da Silva Aguiar, Ivan de Oliveira Gonçalves, and Helio José Coelho‐Junior; data curation, Alejandro Álvarez‐Bustos, Samuel da Silva Aguiar, Isabel Rodríguez‐Sánchez, Ivan de Oliveira Gonçalves, Emanuele Marzetti, and Helio José Coelho‐Junior; writing–original draft preparation, Alejandro Álvarez‐Bustos, Samuel da Silva Aguiar, Isabel Rodríguez‐Sánchez, Emanuele Marzetti, and Helio José Coelho‐Junior; and writing–review and editing, Helio José Coelho‐Junior.

## Funding

Open access publishing was facilitated by Universita Cattolica del Sacro Cuore, as part of the Wiley–CRUI‐CARE agreement.

## Ethics Statement

This study was approved by the Research Ethics Committee of the University of Mogi das Cruzes and was developed in accordance with the Declaration of Helsinki of the World Medical Association (1964, as revised in 1975, 1983, 1989, 1989, 1996, and 2000) and according to Resolution 196/96 of the National Health Council. All participants provided informed consent prior to enrollment.

## Conflicts of Interest

The authors declare no conflicts of interest.

## Data Availability

The datasets generated and analyzed during the current study are available from the corresponding senior authors upon reasonable request and subject to agreement.
